# Association between trajectories of fasting plasma glucose and risk of osteoporosis in non-diabetic and diabetic populations

**DOI:** 10.3389/fpubh.2022.960928

**Published:** 2022-11-08

**Authors:** Ping Wang, Yuanfeng Zhang, Ruiqi Shan, Jing Wu, Sailimai Man, Yuhan Deng, Jun Lv, Xiaona Wang, Jianchun Yin, Yi Ning, Bo Wang, Liming Li

**Affiliations:** ^1^Department of Epidemiology and Biostatistics, School of Public Health, Peking University Health Science Center, Beijing, China; ^2^Department of Statistics and Information, Beijing Center for Disease Prevention and Control, Beijing, China; ^3^Department of Medical Innovation, China Science and Technology Development Center for Chinese Medicine, Beijing, China; ^4^Department of Evidence Based Medicine, Meinian Institute of Health, Beijing, China; ^5^Peking University Health Science Center, Meinian Public Health Institute, Beijing, China; ^6^Department of Social Medicine and Health Education, School of Public Health, Peking University, Beijing, China; ^7^Department of Noncommunicable Diseases, Peking University Center for Public Health and Epidemic Preparedness & Response, Beijing, China; ^8^Beijing MJ Health Screening Center Co., Ltd., Beijing, China; ^9^School of Public Health, Hainan Medical University, Hainan, China

**Keywords:** osteoporosis, diabetes, fasting plasma glucose levels, trajectory analyses, physical examination

## Abstract

**Introduction:**

Previous studies based on a single measure of fasting plasma glucose (FPG) showed an inconsistent conclusion about the association between FPG and osteoporosis risk. Not accounting for time-varying and cumulative average of FPG over time could bias the true relation between FPG and osteoporosis. Our study aims to investigate the association between the trajectories of FPG and osteoporosis risk for non-diabetic and diabetic populations.

**Methods:**

A total of 18,313 participants who attended physical examinations during 2008–2018 were included. They were free of osteoporosis at their first physical examination and followed until their last physical examination before December 31, 2018. We recorded their incidence of osteoporosis and at least three FPG values during follow-up. Their longitudinal FPG trajectories were identified by the latent class growth analysis model based on the changes in FPG. Multivariable logistic regression models were used to analyze the association between the trajectories of FPG and osteoporosis diagnosed in the follow-up physical examination in both non-diabetics and diabetics.

**Results:**

There were 752 incident osteoporosis among 16,966 non-diabetic participants, and 57 incident osteoporosis among 1,347 diabetic participants. Among non-diabetics, the elevated-increasing FPG trajectory was negatively associated with osteoporosis risk in women (odds ratio (OR), 0.62; 95% confidence interval (CI), 0.43–0.88). Premenopausal women with elevated-increasing FPG trajectory had lower osteoporosis risk than those women with normal-stable FPG trajectory (OR, 0.41; 95% CI, 0.20–0.88), while this association was insignificant in postmenopausal women. Among diabetics, those whose longitudinal FPG is kept at a very high level had the highest risk of osteoporosis (OR, 3.09; 95% CI, 1.16–8.22), whereas those whose FPG starts with the high level and keeps on increasing did not exhibit a significantly increased risk (OR, 1.75; 95% CI, 0.81–3.76) compared with those who keep stable moderate-high level of FPG, except in men (OR, 2.49; 95% CI, 1.02–6.12).

**Conclusion:**

Distinct trajectories of FPG are associated with differential risk of osteoporosis in non-diabetic and diabetic populations. Controlling a proper FPG level in different populations is necessary for osteoporosis prevention.

## Introduction

Diabetes, particularly type 2 diabetes mellitus (T2DM), is a common metabolic disease with an increasing disease burden throughout the world (1). People with T2DM had a 40–70% higher risk of hip fragility fracture than normoglycemic individuals (2, 3). Osteoporosis is the main cause of fragility fracture, which is characterized by low bone mass and structural deterioration of the bone tissue. However, previous investigations did not achieve any agreement about the relationship between T2DM and osteoporosis. They have reported that people with prediabetes or T2DM may have higher, lower, or comparable bone mineral density (BMD) values compared with those with normal glucose level (4–6). Fasting plasma glucose (FPG) is the key indicator for defining diabetes and evaluating glucose homeostasis, which is not only affected by various cytokines secreted by bone, but also regulates the differentiation and maturation of osteoblasts (7). Therefore, assessing the association of FPG and the risk of osteoporosis may be helpful in understanding the effect of abnormal glucose metabolism on osteoporosis.

Some (8–11) but not all studies (12, 13) showed that higher FPG was associated with higher BMD in non-diabetes and diabetes. Park et al. (9) showed that elevated baseline FPG (>88 mg/dL in men and >97 mg/dL in women) was associated with the decreased risk of osteoporosis without considering diabetes. While Wang et al. demonstrated that the higher the degree of insulin resistance, the greater the risk of osteoporosis and the association was non-linear (12). It is worth noting that the majority of previous studies on this topic were based on a single measure of FPG, failing to take into account the potential effect of change in FPG concentrations over time. FPG often shows different trends with the change of lifestyle and the use of hypoglycemic drugs. Not accounting for time-varying and cumulative average of FPG over time could bias the true relation between FPG and osteoporosis. Furthermore, osteoporosis developed over a long time. Studies that evaluate the effects of long-term FPG trajectory patterns on osteoporosis are essential.

Therefore, our study aimed to identify subgroups of participants with similar trajectories and to compare the risk of osteoporosis among different trajectories. We examined the potential impact of FPG trajectories, based on repeated longitudinal assessments of FPG from 2008 to 2018, on the risk of osteoporosis in 18,313 non-diabetic and diabetic participants.

## Methods

### Study participants

**Figure 1:**
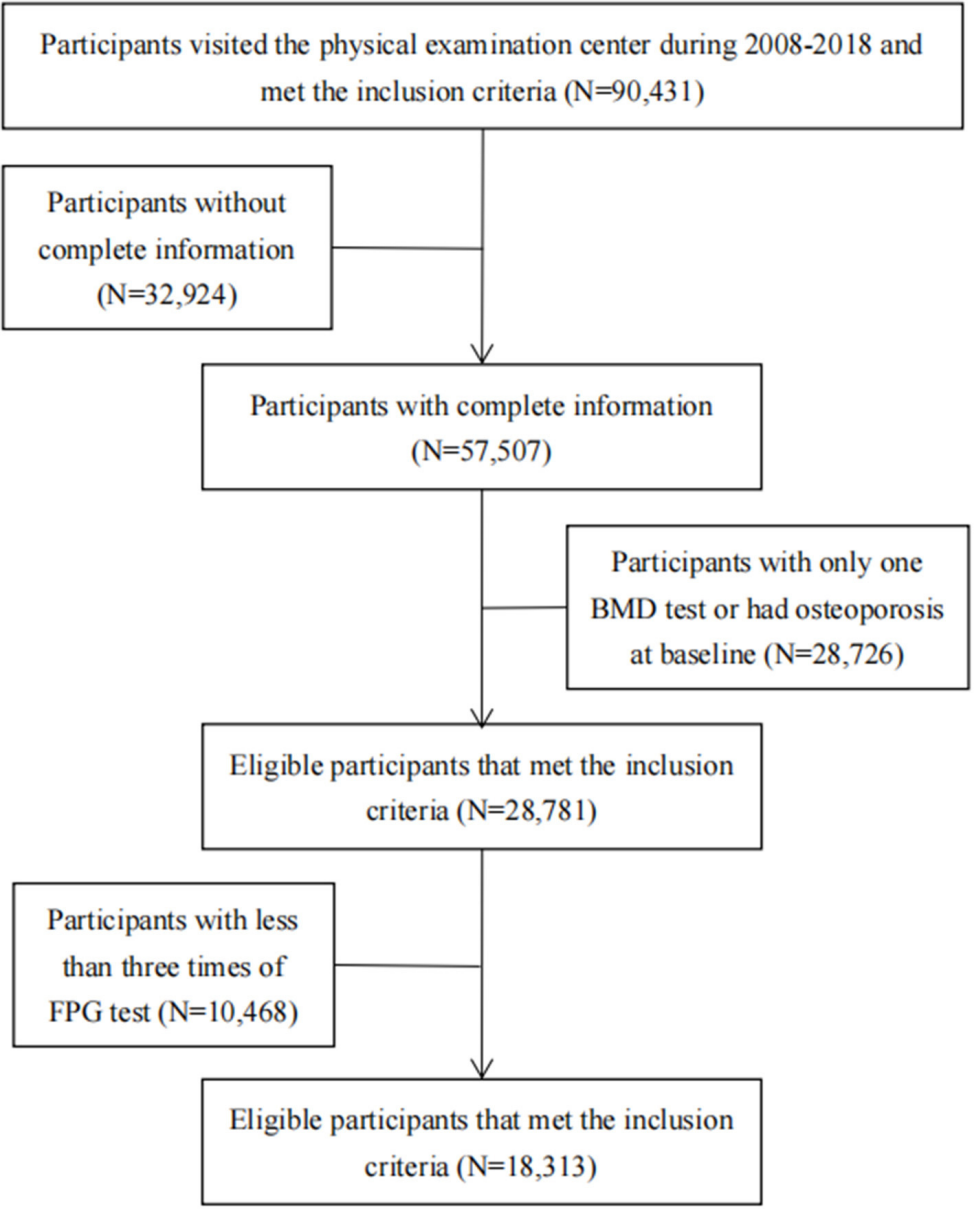
Flowchart of the study.

This study used data from the physical examination of the Beijing MJ Health Screening Center during 2008–2018. Participants visited the center periodically and underwent a series of medical examinations at each visit, including anthropometric measurements, blood tests, urinary tests, imaging tests, and answered a standard self-administered questionnaire. The inclusion criteria in our study were as follows: (1) Age 18 years and above. (2) Should have completed the physical examination and health questionnaire. (3) Should have participated in BMD testing. Then, we excluded individuals with incomplete information (education, occupation, income, smoking, drinking, body mass index (BMI), exercise intensity, calcium supplement, milk, dairy products, BMD, FPG, glomerular filtration rate, etc.) and those without at least two BMD tests. Participants who had already been diagnosed with osteoporosis at their first BMD test were also excluded. To describe the trajectory of FPG, we further excluded participants who were with less than three times of FPG test. Finally, 18,313 participants were included in our study (16,966 non-diabetics and 1,347 diabetics). The research flowchart is shown in Figure 1. Among the total population (*N* = 90,431), the participants included in the final analysis were younger, more male, and more educated than the excluded participants. The differences were statistically significant (Supplementary Table 1).

We defined the participants' first visit to the screening center with the BMD examination as the study baseline and followed them until their last physical examination before December 31, 2018. For individuals with multiple physical examination results in one year during follow-up, we sorted their records by physical examination time and retained the last record containing BMD results in that year.

The study was approved by the Institutional Review Board of Peking University Health Science Center (approval ID: IRB00001052-19077). Our analyses used anonymous data, and the individual informed consent was waived.

### Assessment of FPG and diagnosis of diabetes

An 8-h overnight fasting blood sample was taken from each participant in the morning, and it was measured using the hexokinase method (Cobas 8000 c701 modular analyzer, Roche Diagnostics, or others approved by authorities). After the baseline assessment at the first visit, participants visited the physical screening center annually and measured their FPG. If the measurement was kept for more than once a year, only the last measurement of this year was taken. We followed the participants from their baseline time to their last physical examination before December 31, 2018. The FPG trajectory patterns were identified based on at least three FPG results collected during the follow-up period.

Hemoglobin A1 (A1c) can reflect the mean level of long-term glucose, while FPG has high variability. Only a fraction of all participants had their A1c measurement in our study. In order to ensure the FPG measurement taken once a year for at least three years can be considered to represent the change in the participants' long-term fasting glucose levels in our study, we analyzed the relationship between FPG and A1c, and found that FPG was highly correlated with A1c. The correlation coefficient among 4,436 participants who had an A1c test result is 0.79. We also found a linear relationship between FPG and A1c through scatter plot. *R*^2^ of the linear regression model is 0.62.

Diabetes was defined referring to the American Diabetes Association criteria (14) as (1) FPG ≥7 mmol/L or (2) self-reported physician diagnosis of diabetes or use of anti-diabetic medication at baseline. All diabetic participants were adults without previously reported diagnosis of diabetes, therefore most of them were T2DM.

### Assessment of osteoporosis

The dual-energy X-ray absorptiometry (DXA) examination was used to measure lumbar spine BMD (GE-Lunar Prodigy Advance DXA machine or a Hologic Discovery DXA machine) by technologists in the physical screening center. The instruments were calibrated periodically. During follow-up, a BMD reduction equal to and greater than 2.5 standard deviations of the peak bone mass of normal adults of the same sex and race was considered to be osteoporosis (15).

### Assessment of covariates

Demographic and socioeconomic characteristics, lifestyle factors (smoking status, alcohol consumption, physical activity, milk, dairy products' consumption, and calcium supplements), and female menstrual state were reported by participants using standard self-administered questionnaires at baseline. Height and weight were measured with participants wearing light indoor clothing without shoes during the physical examination. BMI was calculated by dividing weight by height squared. Cutoffs of BMI were chosen according to the well-established criteria for Chinese (16).

Serum creatinine levels were measured by an enzymatic method (Cobas 8000 c701 modular analyzer or Roche Diagnostics). The estimated glomerular filtration rate (eGFR) was calculated by the CKD-EPI (Chronic Kidney Disease Epidemiology Collaboration) equations (17). The participants were staged into categories of renal function based on their eGFR alone as follows: (1) normal eGFR (≥90 ml/min/1.73 m^2^) and without albuminuria, (2) abnormal eGFR with or without albuminuria (< 90 ml/min/1.73m^2^).

### Statistical analysis

#### Classification of the trajectories of FPG

We identified FPG trajectory patterns using Latent Class Trajectory Analysis (LCTA) Model based on the FPG concentrations collected annually from baseline to their last visit time. The LCTA model can help us to estimate the average, variability, and direction of variability simultaneously to investigate the longitudinal changes in FPG levels (18). As the model required a minimum of three time points for a proper model estimation (19), our study included those participants who had at least three times of FPG measures during follow-up. Because non-diabetic and diabetic participants had different FPG levels, we identified the FPG trajectory groups in non-diabetic and diabetic participants separately. Bayesian information criterion (BIC) was used to assess the model fit. We initiated a model with four trajectories and then compared the BIC to that with three, two, and one, respectively. We also took into account the sample size in each trajectory. We compared models and identified the model with two patterns to fit the best in non-diabetes and three patterns in diabetes. Each FPG trajectory was named based on the initial FPG levels and the visual patterns of change in FPG levels. The classification of initial FPG levels was defined as follows: (1) Normal FPG (≤ 5.6 mmol/L), (2) Elevated FPG (5.7–6.9 mmol/L), (3) Moderate high FPG (7.0–8.9 mmol/L), (4) High FPG (9.0–10.9 mmol/L), and (5) Very high FPG (≥11 mmol/L). The patterns of change in FPG were divided into two categories: stable and increasing.

We described the baseline characteristics of non-diabetic and diabetic participants in each trajectory. Data were expressed as n (%) for categorical variables and mean ± standard deviation for continuous variables. The significance of differences in continuous variables between trajectory groups was recorded by the *t*-test, one-way analysis of variance (ANOVA), or the non-parametric test. The categorical variables were estimated by chi-square tests. Participants who developed osteoporosis during the follow-up period were recorded as cases. The crude incidence rate of osteoporosis in each FPG trajectory was calculated as the number of osteoporosis cases reported during the study period divided by the total number of persons at baseline.

### Association between FPG trajectories and osteoporosis

Multivariable logistic regression models were used to investigate the association of FPG trajectories and the risk of osteoporosis, adjusting for potential confounders in four models. Model 1 was adjusted for age, sex, and education. Model 2 was adjusted for all the variables in model 1, and additional variables including smoking status, alcohol consumption, physical activity, milk, dairy products' consumption, calcium supplements, and renal function in non-diabetics. We also adjusted diabetes medication in diabetics. As obesity is correlated with FPG, we built model 3 to adjust all the variables in model 2 and BMI. Model 4 adjusted all the variables in model 3 and baseline BMD. We also conducted a subgroup analysis by sex among non-diabetic and diabetic participants since sex difference in osteoporosis risk was established. To explore the influence of menopause on the relationship between FPG trajectories and osteoporosis risk, we also performed stratified analyses within female group by menopause status.

All statistical analyses were performed using SAS 9.4 for windows (SAS Institute Inc, Cary, NC). The association was regarded as statistically significant at a two-tailed test level of *P* < 0.05.

## Results

A total of 18,313 participants attended the physical examinations. Two trajectories of change in FPG levels were identified during a more than 4-year follow-up period in non-diabetics and three trajectories of FPG in diabetic participants. The median follow-up time was 4.37 years (interquartile range (IQR): 3.06–6.14 years) among non-diabetics, and 4.21 years (IQR: 3.03–5.90 years) in diabetics. The mean age was 42.58 ± 10.31 years old for non-diabetics, and 52.68 ± 9.99 years old for diabetics at baseline. We observed that 752 participants without diabetes (*N* = 16,966) developed osteoporosis, and 57 participants with diabetes (*N* = 1,347) developed osteoporosis. Diabetics were more senior and had lower education levels, higher BMI, and higher percentage of current smokers and drinkers than non-diabetic participants. Participants in non-diabetic and diabetic groups with higher longitudinal FPG levels were also fatter, less educated, and had an unhealthier lifestyle than participants with the lower longitudinal FPG level (Table 1).

**Table 1:** Baseline characteristics in non-diabetic and diabetic participants ^a^.

**Non-diabetes**	**Total**	**Trajectories**		***P* ^a^**	**Diabetes**	**Total**	**Trajectories**			***P* ^a^**
		**Normal-stable**	**Elevated-increasing**				**Moderate-high stable**	**High-increasing**	**Very high-stable**	
No.	16,966	11,582	5,384		No.	1,347	940	307	100	
Case (*n*, %)	752 (4.43)	494 (4.27)	258 (4.79)	0.12	Cases (*n*, %)	57 (4.23)	32 (3.40)	15 (4.89)	10 (10.00)	0.01
Median follow-up years	4.37 (3.06,6.14)	4.50 (3.08,6.28)	4.20 (3.02,6.00)	< 0.01	Median follow-up years	4.21 (3.03,5.90)	4.25 (3.00,6.00)	4.29 (3.12,5.70)	4.03 (2.89,5.30)	0.26
Median visits times	4 (3,6)	4 (3,6)	4 (3,5)	< 0.01	Median visits times	4 (3,5)	4 (3,5)	4 (3,5)	4 (3,5)	0.72
Female (*n*, %)	7,731 (45.57)	6,239 (53.87)	1,492 (27.71)	< 0.01	Female (*n*, %)	317 (23.53)	230 (24.47)	64 (20.85)	23 (23.00)	0.43
Age, year	42.58 ± 10.31	40.54 ± 9.87	46.97 ± 9.87	< 0.01	Age, year	52.68 ± 9.99	53.30 ± 9.99	51.45 ± 10.22	50.62 ± 8.72	< 0.01
Education level (≥Undergraduate) (*n*, %)	12,649 (74.55)	9,023 (77.91)	3,626 (67.35)	< 0.01	Education level (≥Undergraduate) (*n*, %)	708 (52.56)	509 (54.15)	155 (50.49)	44 (44.00)	0.11
BMD at baseline (T score)	0.30 (-0.60,1.30)	0.30 (-0.60,1.20)	0.30 (-0.70,1.40)	0.70	BMD at baseline (T score)	0.20 (-0.70,1.40)	0.30 (-0.70,1.40)	0.20 (-0.60,1.20)	−0.15 (-1.20,0.90)	0.08
BMI, kg/m^2^	24.00 ± 3.46	23.23 ± 3.28	25.67 ± 3.26	< 0.01	BMI	26.27 ± 3.25	26.15 ± 3.18	26.51 ± 3.31	26.58 ± 3.68	0.16
Current smoker (*n*, %)	3,981 (23.46)	2,230 (19.25)	1,751 (32.52)	< 0.01	Current smoker (*n*, %)	528 (39.20)	343 (36.49)	139 (45.28)	46 (46.00)	0.01
Current drinker (*n*, %)	4,223 (24.89)	2,230 (19.25)	1,993 (37.02)	< 0.01	Current drinker (*n*, %)	512 (38.01)	356 (37.87)	123 (40.07)	33 (33.00)	0.44
≥Moderate exercise intensity^b^ (*n*, %)	5,429 (32.00)	3,667 (31.66)	1,762 (32.73)	0.17	≥Moderate exercise intensity^b^ (*n*, %)	396 (29.40)	282 (30.00)	87 (28.34)	27 (27.00)	0.74
Calcium supplements (*n*, %)	1,941 (11.44)	1,335 (11.53)	606 (11.26)	0.61	Calcium supplements (*n*, %)	154 (11.43)	108 (11.49)	35 (11.40)	11 (11.00)	0.99
≥1 cup of milk/Goat's milk per week (*n*, %)	9,819 (57.87)	6,858 (59.21)	2,961 (55.00)	< 0.01	≥1 cup ^c^ of milk/Goat's milk per week (*n*, %)	755 (56.05)	528 (56.17)	167 (54.40)	60 (60.00)	0.61
≥1 serving ^d^ of dairy products per week (*n*, %)	4,885 (28.79)	3,458 (29.86)	1,427 (26.50)	< 0.01	≥1 serving ^d^ of dairy products per week (*n*, %)	281 (20.86)	194 (20.64)	65 (21.17)	22 (22.00)	0.94
Abnormal renal function (*n*, %)	3,564 (21.01)	2,041 (17.62)	1,523 (28.29)	< 0.01	Abnormal renal function (*n*, %)	531 (39.42)	367 (39.04)	127 (41.37)	37 (37.00)	0.67
Postmenopause (female = 7,731) (*n*, %)	1,205 (15.59)	743 (11.91)	462 (30.97)	< 0.01	Taking diabetes medication (*n*, %)	739 (54.86)	468 (49.79)	192 (62.54)	79 (79.00)	< 0.01
					Postmenopause (female = 317) (*n*, %)	155 (48.90)	122 (53.04)	27 (42.19)	6 (26.09)	0.02

*FPG*, fasting plasma glucose; *BMI*, body mass index.

^a^Baseline characteristics among different FPG groups were compared by the chi-square test for categorical variables and one-way analysis of variance (ANOVA) for continuous variables.

^b^Mild: golf, sweep the floor, mop the floor, calisthenics, etc.; Moderate: basketball, volleyball, table tennis, badminton, etc.; ≥severe: climb stairs, butterfly stroke, etc.

^c^One cup is equivalent to 240 ml of milk, 240 ml yogurt, or 4 tablespoons of milk powder.

^d^One serving is equivalent to 30 g of cheese or 1 slice of cheese.

### Trajectories of FPG

**Figure 2:**
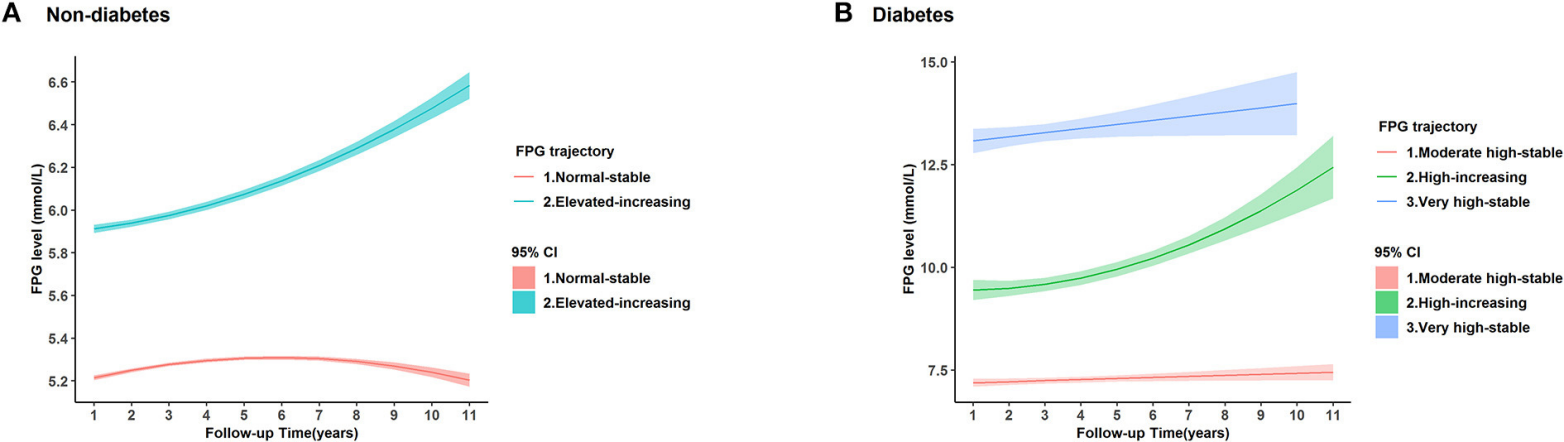
Trajectories of fasting plasma glucose (FPG) in 18,313 individuals based on the latent group membership. The lines are the trajectory of fasting plasma glucose (FPG) over time in **(A)** non-diabetic participants and **(B)** diabetic participants. The shadow was the 95% confidence interval (CI) of each trajectory. We defined that the stable normal level referred to FPG ≤ 5.6 mmol/L, elevated FPG level referred to FPG in 5.7–6.9 mmol/L, moderate-high level referred to FPG in 7.0–8.9 mmol/L, high level of FPG referred to FPG in 9.0–10.9 mmol/L, and very high levels referred to FPG>11.0 mmol/L.

The FPG trajectories in the non-diabetic and diabetic groups were significantly different (Figure 2). There were 68.27% of non-diabetics in the “normal-stable” trajectory group, in which they started with a normal FPG level and then remained stable in the following period; 31.73% of non-diabetics had an elevated level of FPG at baseline and then experienced an increase in FPG level. There were 69.78% of diabetics in the “moderate high-stable” trajectory group, in which they started with moderate-high FPG level and then remained stable all the time; 22.79% of diabetics in the “high-increasing” trajectory group, in which they began with a high FPG level and then experienced an increase in FPG level; 7.42% of diabetes in the “very high-stable” trajectory group, in which they started with a very high FPG level and then kept stable all the time.

### Associations between FPG trajectories and osteoporosis in non-diabetics and diabetics

In non-diabetics, the incidence of osteoporosis in the elevated-increasing trajectory group (4.79%) was slightly higher than those in the normal-stable group (4.27%). But the difference was not significant (*P* = 0.12). While in diabetics, the prevalence of osteoporosis increased with the increase of FPG, and the difference in each trajectory had statistical significance (*P* = 0.01) (Table 1).

Table 2 presents the associations between FPG trajectories and osteoporosis risk in non-diabetic and diabetic participants. In non-diabetic participants, FPG trajectories were significantly associated with osteoporosis when adjusted for potential confounders in model 1 and model 2. Participants in the elevated-increasing trajectory group had a significantly lower risk of osteoporosis than those in the normal (≤ 5.6 mmol/L)-stable group. However, after adjusting for BMI, this trajectory did not exhibit a significantly decreased risk of osteoporosis (OR, 0.90; 95% CI, 0.76–1.06 in model 3). There was gender difference in the association between FPG and osteoporosis risk in non-diabetic participants. The negative association between FPG and osteoporosis risk was statistically significant only in women (OR, 0.62; 95% CI, 043–0.88). We also found that premenopausal women with elevated-increasing FPG trajectory had a lower osteoporosis risk than those with normal-stable FPG trajectory (OR, 0.41; 95% CI, 0.20–0.88). While this association was insignificant in postmenopausal women.

**Table 2:** Odds ratios (ORs) of trajectories of FPG for incident osteoporosis.

**Non-diabetes**	**Trajectories**		**Diabetes**	**Trajectories**		
	**Normal-stable**	**Elevated-increasing**		**Moderate-high stable**	**High-increasing**	**Very high-stable**
Model 1 ^a^	1.00 (reference)	0.74 (0.63–0.88)	Model 1 ^a^	1.00 (reference)	1.58 (0.83–2.98)	3.50 (1.63–7.54)
Model 2 ^b^	1.00 (reference)	0.76 (0.64–0.90)	Model 2 ^b^	1.00 (reference)	1.59 (0.83–3.05)	3.80 (1.69–8.57)
Model 3 ^c^	1.00 (reference)	0.90 (0.76–1.06)	Model 3 ^c^	1.00 (reference)	1.59 (0.83–3.07)	3.87 (1.70–8.81)
Model 4 ^d^	1.00 (reference)	0.87 (0.72–1.04)	Model 4 ^d^	1.00 (reference)	1.75 (0.81–3.76)	3.09 (1.16–8.22)
Male ^d^	1.00 (reference)	1.02 (0.82–1.28)	Male ^d^	1.00 (reference)	2.49 (1.02–6.12)	4.10 (1.28–13.17)
Female ^e^	1.00 (reference)	0.62 (0.43–0.88)	Female ^e^	1.00 (reference)	0.64 (0.06–6.49)	-
Pre-menopausal women ^f^	1.00 (reference)	0.41 (0.20–0.88)				
Postmenopausal women ^f^	1.00 (reference)	0.71 (0.47–1.06)				

*FPG*, fasting plasma glucose; *BMI*, body mass index.

^a^Model 1was adjusted for age, sex, and education.

^b^Model 2 adjusted for variables in model 1, and further adjusted for renal function, smoking status, alcohol consumption, physical activity; milk, dairy products' consumption, and calcium supplements in non-diabetes. Diabetes also adjusted for diabetes medication variable.

^c^Model 3 adjusted for variables in model 2, and further adjusted for BMI.

^d^Model 4 adjusted for variables in model 3, and further adjusted for bone mineral density (BMD) at baseline.

^e^Adjusted factors in model 4 and we further adjusted for baseline menstrual status variables.

^f^Adjusted factors in model 4.

Among diabetic participants, a very high-stable FPG level was positively associated with osteoporosis risk. Participants in this group had 3.09 times higher risk than those in the moderate high-stable trajectory group. Participants in the high-increasing group group did not show significant association with osteoporosis but tended to be positively associated with osteoporosis (OR, 1.75; 95% CI, 0.81–3.76 in model 4). When stratified by sex, the high-increasing FPG trajectory was positively associated with osteoporosis risk in men (OR, 2.49; 95% CI, 1.02–6.12). The very high-stable FPG trajectory was also significantly associated with osteoporosis risk in men (OR, 4.10; 95% CI, 1.28–13.17). As we did not have a sufficient sample size in the female participants, we failed to estimate the association between the very high-stable FPG trajectory and osteoporosis risk in women and did not conduct stratified analysis by menopause status.

## Discussion

In this study, we found that different FPG trajectories in non-diabetic and diabetic populations had apparent different influences on osteoporosis risk. Elevated long-term FPG in non-diabetes women is negatively associated with osteoporosis risk, and a very high long-term FPG level in diabetes is positively associated with osteoporosis risk.

A study by Li et al. (8) reported a significantly decreased prevalence of osteoporosis with blood glucose among people with T2DM and Park et al. (9) found this association existed but ignored the diabetes status. While the study by Wang et al. (12) demonstrated that the higher degree of insulin resistance noted a higher risk of osteoporosis, but it was a non-linear relationship. It might be implied that hyperglycemia was correlated with increased osteoporosis risk. The differences reported in these studies might derive from the single measure of FPG which could not catch the effects of time-varying and cumulative average of FPG on osteoporosis. According to our results, we supposed that there might be a bidirectional association between FPG trajectory and osteoporosis risk. That is to say an appropriate elevated FPG level within the normal range may stimulate bone formation, and an extremely high level of FPG may be harmful to the bone. Candidate genetic locus, such as rs6867040 at the *ITGA1*, influencing both FPG and BMD has been identified (20), partly explaining the genetic contribution to the linking between FPG and osteoporosis. Glucose intake is supposed to be the main regulator of both osteoblastic differentiation and function. Previous studies have demonstrated that glucose is the main nutrient of osteoblasts. A ketogenic diet can reduce the height of children. Raising blood glucose levels can help osteoblasts restore collagen syntheses and initiate bone formation (21, 22). While, high glucose states with insulin resistance can impair the bone formation and lead to diabetic bone disease (7, 23). The higher degree of insulin resistance noted a higher risk of osteoporosis (12). So, our results provided additional information to understand the dynamic FPG and risk of osteoporosis over time. Previously, Kanazawa et al. (24) reported that the improvement of glycemic control in T2DM patients was associated with increased serum osteocalcin levels. The osteoblastic differentiation may be stimulated and bone formation enhanced. Our results supported the importance of glycemic control in the prevention of osteoporosis.

In non-diabetic participants, the median follow-up years in the elevated-increasing FPG trajectory group were shorter than those of the normal-stable FPG trajectory group. It might lead us to fail to observe more outcomes in the elevated-increasing FPG trajectory group, so that the association between FPG trajectory and osteoporosis risk was insignificant. Moreover, the association between FPG trajectory and osteoporosis might also be affected by the BMI. Previous studies had found that there was interaction between fat/glucose metabolism and bone metabolism (24). Obesity may affect both bone and glucose homeostasis (7). The positive relationship between FPG and BMD was probably confounded by the BMI. Therefore, the complex relationship among obesity, FPG, and osteoporosis is worth to conduct further studies to investigate this aspect. In the subgroup analysis, we found that the negative association between FPG trajectory and osteoporosis risk was significant in women. This corresponded with the study conducted by previous studies (9, 25). Moreover, we found the significant association only existed among premenopause women. As menopause is one of the risk factors for osteoporosis in women (26), we supposed that the negative association between FPG and osteoporosis risk was influenced by accelerated bone loss after the menopause, so the association among postmenopausal women was not statistically significant. To our knowledge, no prior studies have examined the potential impacts of glucose trajectories on osteoporosis risk among non-diabetics. This result is worthy of future prospective studies to verify the causal association between them.

In diabetic participants, the participants in the high-increasing FPG group did not show significant association with osteoporosis, except in men. Previous studies have reported that FPG variability may be a novel risk factor for osteoporotic fractures (27, 28). The higher variability of FPG, the higher risk of osteoporotic fractures. This may indicate that FPG variability was also a risk factor associated with osteoporosis. In our study, FPG in the high-increasing group increased from about 9 mmol/L to nearly 12.5 mmol/L. The high FPG variability in this trajectory might account for the positive association between FPG and osteoporosis risk, while the extent was very modest, so that it did not reveal significance at the current sample size. In the subgroup analysis, we found that the association was only significant in men. The result was different from the study conducted by Wang et al. (12). The non-significant result in females in our study might have occurred due to the small sample size. Future studies should expand the sample size to verify these results.

It is worthy of mentioning two strengths of the study. First, the variation of FPG was captured in a large sample size with BMD tests and FPG tests at multiple time points with high quality. Second, the LCTA model is a powerful approach to describe the trajectories of FPG over the years. It does not assume a priori the existence of change in FPG levels in the population but allows distinctive latent FPG trajectories derived from data (18). This method helps us to discover unexpected yet potentially meaningful trajectories that may have otherwise been overlooked in the traditional analysis of exposure factors.

There are also some limitations to our study. First, our findings are based on a cross-sectional study. The nature of this study design can only explore the association between FPG trajectory and osteoporosis risk rather than proving causality between them. Longitudinal studies are needed to confirm the causal relationship between the two. Second, we did not define diabetes according to the hemoglobin value, as a hemoglobin test was not conducted for all participants in our study. But we adopted self-reported diabetes and used antidiabetic medication as one of the criteria to make the classification of diabetes as accurate as possible. A1c reflects the mean level of long-term glucose, while, FPG has high variability. We had analyzed the relationship between FPG and A1c in our study, and found that FPG was highly correlated with A1c. Therefore, we thought that FPG measurements taken once a year for at least three years can be considered to represent the change in the participants' long-term fasting glucose levels. Third, the finally included participants were more likely to be young, male, and educated, compared with the excluded participants. The included participants may introduce potential selection bias, which may weaken the association between FPG and osteoporosis risk. In addition, our study population included Chinese adults attending routine health checkups. This could limit the generalizability of our findings. We should be cautious about generalizing the results to other populations. However, we think there is no reason to expect that underlying mechanisms for longitude very high FPG influence on the bone may be different, our results reveal the general association of them and suggested that more attention should be paid to the dynamic changes of blood glucose in bone health. Fourth, two DXA machines were mainly used to assess BMD in our study. Measurements may vary among instruments from different manufacturers. However, a previous study conducted on the Chinese population has found the difference between manufacturers for BMD to be no more than 2% (29). We thought the differential diagnosis of osteoporosis in our study was minimal. Finally, we did not collect variables including diabetes control status (e.g., duration of diabetes, vascular complications) and detailed medications for diabetes in our questionnaire as part of the routine health examination. Although we had adjusted the use of medications for diabetes in our model, the failure to include detailed medications for diabetes might have biased the results.

In summary, our findings indicate that elevated-increasing FPG level, but still in the normal range, was negatively associated with the risk of osteoporosis among non-diabetic women. Those women with FPG kept in the very high level had the highest osteoporosis risk than those women with stable moderate-high level of FPG among diabetics. Establishing an appropriate longitudinal FPG control standard according to the different associations between FPG and osteoporosis risk in different populations is helpful for the prevention of osteoporosis and control of diabetes.

## Data availability statement

The raw data supporting the conclusions of this article will be made available by the authors, without undue reservation. Requests to access the datasets should be directed to BW, paul@meinianresearch.com.

## Ethics statement

The studies involving human participants were reviewed and approved by Institutional Review Board of Peking University Health Science Center, (approval ID: IRB00001052-19077). The Ethics Committee waived the requirement of written informed consent for participation.

## Author contributions

PW analyzed data, drafted the manuscript, and interpreted the results. BW proposed the analysis plan. RS, JW, YZ, XW, JY, SM, YD, and JL revised the manuscript. YN, BW, and LL conceived the study and finally approved the manuscript and the guarantors of this work. All authors contributed to the article and approved the submitted version.

## Funding

Funding for this article was provided by the National Natural Science Foundation of China (No. 91846303) and the National Key R&D Program of China (Nos. 2020YFC2004703 and 2020YFC2003400). No copyrighted materials were used in this article. Contact the corresponding author for copyrighted survey questionnaire.

## Conflict of interest

Authors XW and JY were employed by the company Beijing MJ Health Screening Center Co., Ltd. The remaining authors declare that the research was conducted in the absence of any commercial or financial relationships that could be construed as a potential conflict of interest.

## Publisher's note

All claims expressed in this article are solely those of the authors and do not necessarily represent those of their affiliated organizations, or those of the publisher, the editors and the reviewers. Any product that may be evaluated in this article, or claim that may be made by its manufacturer, is not guaranteed or endorsed by the publisher.
