# Supplementary material for: Association between trajectories of fasting plasma glucose and risk of osteoporosis in non-diabetic and diabetic populations
Source: Front Public Health. 2022 Nov 8;10:960928. doi: 10.3389/fpubh.2022.960928 (PMC9679646; doi:10.3389/fpubh.2022.960928)
Supplement: Supplementary file 1 [file Table_1.docx]

**Supplementary Table 1 Baseline characteristics in excluded and included participants**

|  | Total population(N=90431) | | |  | Dynamic cohort(N=28781) | | |
| --- | --- | --- | --- | --- | --- | --- | --- |
|  | Exclusion  (N=72118) | Inclusion  (N=18313) | *P* |  | Exclusion  (N=10468) | Inclusion  (N=18313) | *P* |
| Sex(N,%) |  |  |  |  |  |  |  |
| Male | 38385(53.23) | 10265(56.05) | <0.01 |  | 5819(55.59) | 10265(56.05) | 0.45 |
| Female | 33733(46.77) | 8048(43.95) |  |  | 4649(44.41) | 8048(43.95) |  |
| Age | 44.96±12.12 | 43.32±10.62 | <0.01 |  | 43.52±10.69 | 43.32±10.62 | 0.13 |
| Educational level |  |  |  |  |  |  |  |
| High school and below | 10309(16.3) | 1427(7.79) | <0.01 |  | 1164(11.12) | 1427(7.79) | <0.01 |
| Technical secondary school or junior college | 13590(21.5) | 3529(19.27) |  |  | 2197(20.99) | 3529(19.27) |  |
| Undergraduate | 24516(38.9) | 8202(44.79) |  |  | 4324(41.31) | 8202(44.79) |  |
| Master degree or above | 14682(23.3) | 5155(28.15) |  |  | 2783(26.59) | 5155(28.15) |  |
